# Beta diversity subcomponents of plant species turnover and nestedness reveal drivers of community assembly in a regenerating subtropical forest

**DOI:** 10.1002/ece3.70233

**Published:** 2024-09-16

**Authors:** Coskun Guclu, Chung‐Lim Luk, Louise Amy Ashton, Sawaid Abbas, Michael J. W. Boyle

**Affiliations:** ^1^ Ecology and Biodiversity Department, School of Biological Sciences The University of Hong Kong Hong Kong Hong Kong SAR; ^2^ Department of Land Surveying and Geo‐Informatics The Hong Kong Polytechnic University Hung Hom Hong Kong SAR

**Keywords:** community assembly, nestedness, secondary succession, sub‐tropical forests, turnover

## Abstract

Secondary forests represent a significant proportion of global forest cover, with over 70% of forests in East Asia classified as regenerating. While succession has been studied extensively in temperate systems, trajectories of subtropical succession remain poorly characterized in highly disturbed, urban‐adjacent forests. Investigating the additive beta diversity components of turnover and nestedness may reveal community assembly mechanisms driving secondary succession. The present study investigates plant community assembly along a successional gradient from 7 to 70 years following the onset of succession in secondary subtropical forests in Hong Kong, China. Plant survey data for 28 plots were analysed, generating additive Simpsons turnover and nestedness beta diversity metrics. Dissimilarity matrices were generated and modelled as a function of environmental matrices including forest plant community age (years following onset of secondary succession), inter‐community distance (metres), and soil moisture saturation (%) across three elevational bands using generalized dissimilarity models. Nonmetric multidimensional scaling of plant communities was conducted with Bray–Curtis dissimilarity matrices. Inter‐community distance and successional age differentially influenced plant species turnover between lowland and Montane forest types. Models of nestedness found that plot age and soil moisture saturation were significant drivers of nestedness patterns in plant communities across elevational classes. Turnover represented a higher proportion of Sorensen beta diversity than nestedness, while ANOSIM found significant differentiation between plant communities at different successional stages. Turnover patterns suggest a deterministic model of community assembly, with strong patterns of species replacement between communities at fine spatial scales and successional stages, as well as clear compositional shifts between lowland and montane forest types. NMDS analysis and functional compositional assessments suggested a transition from early successional communities with a high proportion of shrub species, to later successional communities with a higher proportion of tree species, with an increase in species turnover with greater age dissimilarity.

## Graphical abstract

Secondary forests represent significant proportions of global forest cover, and an increasing share of global forests are undergoing secondary succession. The present study investigated drivers of plant community assembly in secondary forests in sub‐tropical Hong Kong. Forests were found to be influenced significantly by age since onset of succession, but this was determined by elevational gradient position.

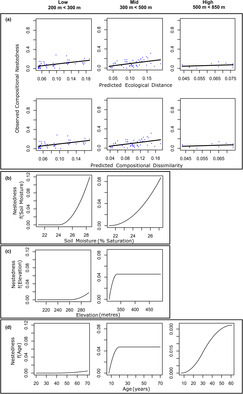

## 1 INTRODUCTION

Secondary forests are integral for the conservation of biodiversity and the fulfilment of environmental targets, with international initiatives such as the United Nations Environmental Programme, and the China National Biodiversity Action Plan targeting the restoration of millions of hectares of forest by 2030 (Meli et al., 2017; UNEP, 2022). Forest ecosystems offer a range of ecosystem services and host a quarter of Earth's biodiversity (Nichol & Abbas, 2021; Pan et al., 2011; Pei et al., 2010; Yarwood et al., 2020). However, disturbances to forest ecosystems from industrialization, urbanization, agriculture and silviculture have caused historical deforestation in the tropics and sub‐tropics of South China, modifying plant community species composition and compromising vital ecological functions and services (Abbas et al., 2019; Måren et al., 2017; Purschke et al., 2017; Zhuang & Corlett, 1997). Over 80% of global forests have undergone or are currently subject to degradation and fragmentation due to human disturbances, and determining methods for forest conservation and restoration is a major concern for the fulfilment of environmental targets (Chazdon, 2003; Collins et al., 2017; Holl et al., 2013; Watson et al., 2018).

Anthropogenic disturbances to forest ecosystems have led to the creation of subjective categories which describe forests as either primary, secondary or disturbed, the classification of which is subject to contextual, spatial and temporal patterns of disturbance, and the idiosyncrasies of regional land use history (Chen et al., 2016; Chokkalingam & De Jong, 2001; Turner et al., 1997). Primary forests are described as undisturbed by human activity or from significant natural disturbances and typically host the highest levels of biodiversity (GFO, 2020; Mang & Brodie, 2015). Conversely, secondary forests regenerate following the clearance of primary forest by either human or natural disturbances, and secondary succession proceeds from extremely small remnants, dispersal or seed bank (Chokkalingam & De Jong, 2001). Disturbed forests are distinct from secondary forests due to their sustained exposure to anthropogenic influences, such as selective logging in Malaysian Dipterocarp forests, or rubber plantation in South China, which continue to impact the forest plant community structure and composition (Ewers et al., 2024; Ma et al., 2019; Saiful & Latiff, 2014).

Although tropical and sub‐tropical forests have been structurally and compositionally altered by humans since the Pleistocene, the recent impacts of human activity following the onset of the industrial revolution and the proliferation of European colonialism from the 19th century onward have altered the global proportion of primary and secondary forest in the Asian tropics and subtropics (Barlow et al., 2007; Gibson et al., 2011; Phillips et al., 2017; Roberts et al., 2021). In East Asia, 78% of forests are classified as regenerating or secondary (GFO, 2020). Forest plantation has also drastically increased in the region, rising by 138% between 1990 and 2020, which is largely due to the major afforestation programmes conducted by the Chinese government from 2000 to 2020 (Nichol & Abbas, 2021). Within the Southern Chinese provinces of Guizhou, Guangxi and Yunnan alone, secondary forests undergoing succession may represent as much as 70% of total forest cover, while in Hong Kong secondary forests dominate forest cover alongside scattered remnant patches and Feng Shui woodlands (Dudgeon & Corlett, 1994; Zhuang & Corlett, 1997; Li et al., 2022; Lee et al., 2023). With secondary forests now representing a major share of regional forest cover, determining the time frames for secondary succession to form plant communities comparable to primary forests, or evaluating whether this is possible without human intervention, represents a major conservation priority for the 21st century (Ewers et al., 2024). In cases where primary forest has been completely cleared over sustained time periods such as large parts of East Asia, exploring the mechanisms through which new communities assemble during secondary succession may help to outline conservation and restoration priorities (Corlett, 2011).

A global review of temperate and tropical forests systems previously suggested a range of a 20–60 years period for regenerating secondary forest to resemble a primal compositional and structural state, while analysis of tropical forest recovery across South America and Africa posited 120 years as a minimum period for regeneration (Bowen et al., 2007; Poorter et al., 2021). These syntheses suggested that recovery in Asian tropical forests may be slower than in South America and Africa, yet study sites in East‐Asian monsoonal forests, particularly in subtropical South China, were under‐represented (Cole et al., 2014). Additionally, these investigations did not integrate topographic and spatial gradient effects alongside forest age. Such gradients may significantly impact temporal patterns of plant community change during secondary succession due to the influences these variables have upon community assembly mechanisms.

Community assembly theory offers a mechanistic understanding of the dispersal, environmental and biotic filters which plant species must pass through to form forest communities during secondary succession (Vellend, 2016). Dispersal filters represent the geographic limits of a species' range. Plant community composition during early succession is limited to species already present in remnant patches, or species able to disperse from proximal or distant regions, and those able to germinate from the seed bank if one remains intact (Dudgeon & Corlett, 2011; Laurance, 1999). If dispersal networks are functional, or if small‐seeded, wind‐distributed plant species are regionally present, colonization of cleared land may be swift (Corlett, 2011; Mayhew et al., 2019). However, degradation of dispersal networks such as extirpations of dispersing frugivore and scatter‐hoarding fauna can significantly limit the pace and extent of forest recovery, particularly for later successional plant species with larger, or non‐fleshy fruits (Corlett, 2011; Escribano‐Avila et al., 2018; Galetti et al., 2013). For plant species able to pass through dispersal filters and environmental filters, the abiotic parameters of a locality such as temperature, precipitation, and the biochemical and geophysical quality of strata determine physiological limits for the range of species that can survive. During early succession, fast‐growing shrub species may be preferentially selected due to their tolerance to the harsh abiotic conditions in the absence of a closed canopy and dry, nutrient‐poor soil (Abbas et al., 2019; Horn, 1974; Odum, 1969; Prach & Walker, 2019). Abiotic conditions may also vary across topographic gradients such as elevation, where temperature can vary greatly, leading to differentiated selective regimes. Finally, biotic filters such as intra‐ and inter‐specific competition, as well as facilitative and mutualistic interactions, further limit the composition of the local species pool at different times during secondary succession (Vellend, 2010). As a canopy begins to form, early successional shrub species may be shaded out by slower‐growing larger tree species, with competition for light and soil nutritive resources becoming a prominent determinant of community composition (Horn, 1974; Odum, 1969; Prach & Walker, 2019). Integrating temporal, topographic and spatial gradients can therefore help to determine the status and trajectories of forest plant communities during secondary succession.

While analysing plant species richness and diversity at the local level (alpha diversity) can be informative (Abbas et al., 2019), assessment of inter‐site variation in species composition (beta diversity) can delineate the relative contribution of community assembly filters to community formation during secondary succession (Baselga, 2010; Batista et al., 2021; Murphy et al., 2015). For instance, communities exhibiting dispersal‐based filtering may have steeper rates of dissimilarity with increasing geographical distances between communities (Batista et al., 2021; Dobrovolski et al., 2011) while variations in topography and microclimate create differential selective regimes, which sort species along environmental gradients (Oikonomou & Stefanidis, 2020). Additive forms of Beta diversity such as the Sorenson (*β*_Sor_) beta diversity metric can be partitioned into species nestedness and turnover, which quantify species loss or gain and replacement respectively (Baselga, 2010). The responses of these beta diversity (*β*) metric sub‐components to environmental gradients may reflect the contribution of niche (deterministic) and neutral (stochastic) processes driving community assembly during secondary succession (Soininen et al., 2018). Turnover (species replacement) can indicate sorting by competition, dispersal and environmental filtering, while nestedness (species loss or gain) can reflect extinction‐colonization dynamics from communities of high species richness to communities of low species richness (Baselga, 2010; Si et al., 2016). Assessing the variation of plant species turnover and nestedness along temporal, spatial, topographic and environmental gradients may identify processes shaping plant communities. These metrics may be highly informative for conservationists in cases where turnover outweighs nestedness, which may indicate that a large number of sites should be conserved in order to conserve the highly differentiated communities with many rare species in a landscape, or where spatial turnover is steep, suggesting dispersal limitations (Oikonomou & Stefanidis, 2020).

Previous works assessing beta diversity in secondary tropical forest ecosystems have found contradicting patterns of beta diversity metrics in response to forest age. While Villa et al. (2021) found that turnover of plant species increased with increasing age in tropical forests in Venezuela, (López‐Martínez, Hernández‐Stefanoni, et al., 2013) found that turnover decreased with time since onset of secondary succession in Mexican tropical forests. These studies were conducted on different successional age gradients, with either no or narrow elevational gradients and no integration of spatial or environmental variables. Plant communities assemble across multiple environmental gradients which may alter the influence of community age. Elevation can significantly influence plant community composition through altering abiotic conditions, and accounting for the influence of elevation is thus crucial for accurately parsing the effects of age on plant community assembly in tropical and subtropical forest secondary succession (Descombes et al., 2017; Willig et al., 2011). Inter‐community distance may also significantly influence beta diversity by disproportionately influencing dispersal and colonization‐extinction mechanisms reflected in strong responses of turnover and nestedness respectively to increasing distance between communities (Batista et al., 2021; Oikonomou & Stefanidis, 2020).

This is crucial to understanding the trajectories of secondary forest succession and is key to implementing effective conservation and restoration initiatives, as findings may indicate the capacity of forests to regenerate spontaneously, indicate the number of communities requiring official protection, and may justify additional conservation measures such as transplantation, assisted migration and reintroductions of seed and fruit‐dispersing species (Arroyo‐Rodríguez et al., 2017). The exploration of plant species turnover and nestedness dynamics during secondary succession remains understudied in tropical East Asia, particularly in South China. While studies of secondary succession in subtropical forests in Jiangxi (Zhang et al., 2021), Hunan (Liu et al., 2022) and Guangdong (Han et al., 2023) have previously explored patterns of local changes in species, functional and phylogenetic diversity, these investigations have not investigated the explicit responses of beta diversity metrics during secondary succession (Abbas et al., 2019). Such investigations are pertinent to regional conservation, and in regions such as Hong Kong with documented forest chrono‐sequences, the responses of beta diversity during secondary succession can be more extensively explored.

The subtropical forests of Hong Kong have been subject to extensive historical disturbances through logging, agriculture and urbanization over several centuries, and have been undergoing spontaneous and assisted secondary succession following almost complete clearance in the 1940s (Fischer & Zhang, 2016; Xing et al., 1999; Zhu et al., 2023; Zhuang & Corlett, 1997). Natural recovery from remnant patches, old‐growth refugia in remote locations and the intensive plantation of native and exotic species following 1945 have led to the formation of the present regional floristic species pool (Abbas et al., 2019; Corlett, 1999; Fischer & Zhang, 2016). Dispersal from old managed ecosystems such as Feng Shui Woodlands is unlikely to have significantly contributed to this process as local fruit‐dispersing networks were severely degraded following the widespread extirpation of native dispersing fauna (Chung & Corlett, 2006; Corlett, 2011; Corlett & Hau, 2000; Zhuang & Corlett, 1997). The community assembly filters shaping secondary succession of Hong Kong's tropical forests remain unclear and require further investigation, offering opportunities for understanding the influence of temporal, topographic, spatial and environmental gradients in shaping subtropical forest plant community turnover and nestedness following periods of protracted disturbance.

The present study aimed to examine the relative importance of successional age, elevation and inter‐community distance in shaping patterns of plant species' turnover and nestedness along these gradients in regenerating secondary forests in Hong Kong. The present study addresses the following questions: (i) How do temporal variables of forest age, topographical variables of elevation and spatial variables of inter‐community distance influence beta diversity subcomponents of turnover and nestedness, and which of these are primary in shaping these aspects of regional diversity? and; (ii) Which plant species assemblages are associated with secondary forest age groups? In order to address these questions, plant species presence–absence data were used to conduct a beta diversity analysis across three elevational gradients at low lowland, mid‐level lowland and montane forest types. The following hypotheses were tested; (H1) Species turnover will increase with increasing forest age dissimilarity due to the abiotic and biotic changes that occur during secondary succession leading to differentiated selective regimes and a transition from early‐ to late‐stage successional plant species, as well as shifts from shrub dominance in early succession to tree dominance in late succession; (H2) Species turnover will increase sharply with inter‐community distance due to the dispersal limitations unique to Hong Kong (Corlett, 2011) and; (H3) Species turnover will be higher between lowland and montane forest biomes due to the differentiation of species with elevational niche. This is due to the seasonal temperature flux in Hong Kong which has led to the characterization of the regional climate as sub‐tropical rather than tropical, which can lead to periodic frosts forming and winter low temperatures below 10°C at elevations above 500 m, presenting a significant abiotic filter which makes occupation of uplands untenable to several local plant species which are typically confined to lowland areas below 500 m (Corlett, 1999; Dudgeon & Corlett, 1994; Dudgeon & Corlett, 2011).

## 2 METHODS

**FIGURE 1:**
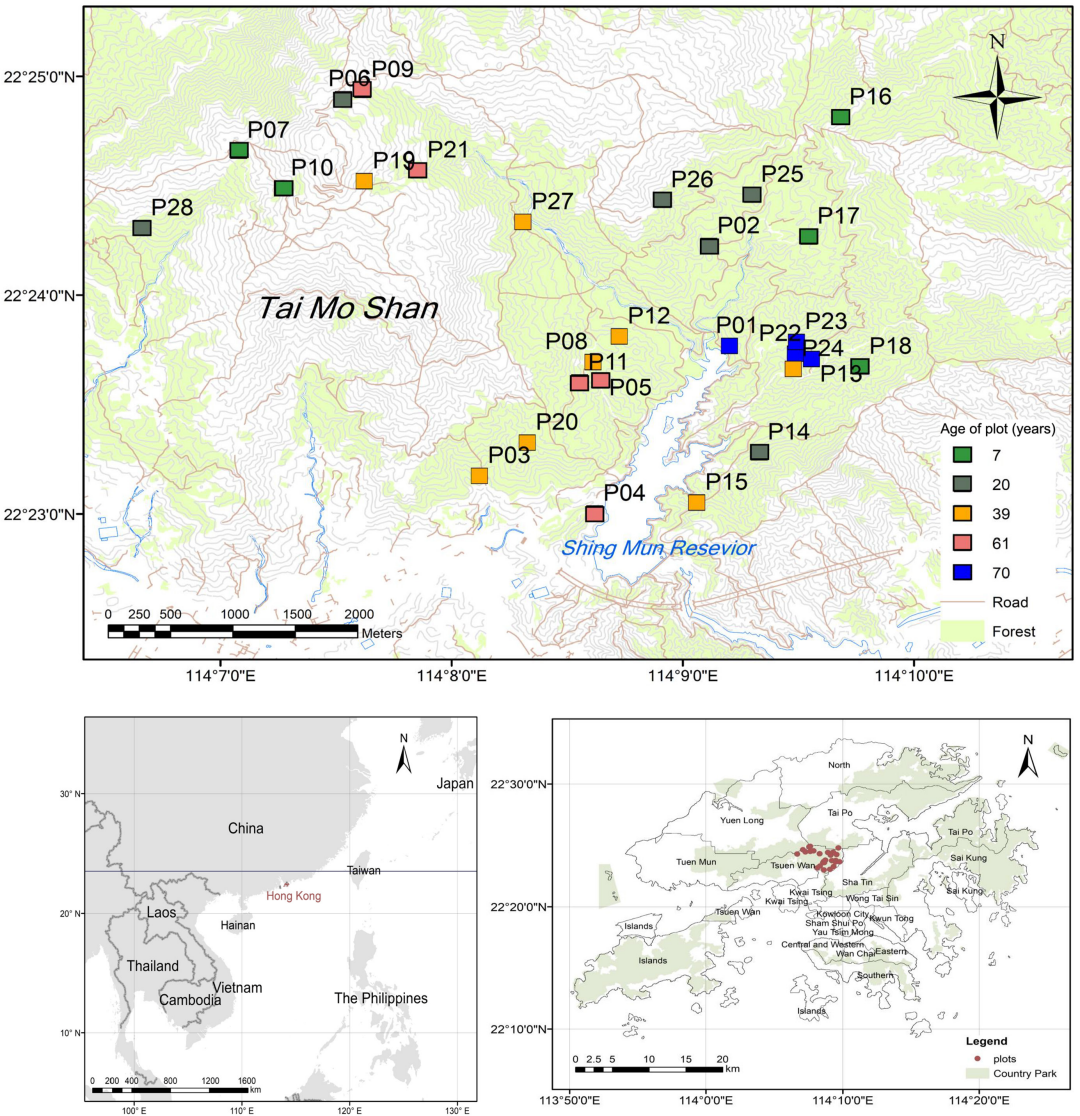
Study sites located within the Tai Mo Shan and Shing Mun Country Park in Hong Kong SAR.

The study was conducted along a secondary forest successional gradient in the Tai Mo Shan and Shing Mun Country Parks in Hong Kong. Hong Kong has distinctive wet, hot summers from May to October with mean daily temperatures of 24–33°C and dry, cool winters from November to April with mean daily temperatures of 9–22°C. The mean annual rainfall is 1800‐2100 mm (Dudgeon & Corlett, 2011). The secondary forests in the present study developed through spontaneous succession following extensive deforestation in World War II. Twenty‐eight 20m^2^ plots within these regenerating sites were characterized by (Abbas et al., 2019) (Appendix S1; Figure 1), using historical ariel photographs to designate time since onset of succession from open grassland. The plots varied in average age, with the youngest age class at 7 years from onset of succession from open grassland, and the oldest over 70 years. Distances between plots were calculated by generating matrices of distance between plot edges, and inter‐plot distance ranged from 100 to 5459.41 m. Plot elevations ranged from 205.1 to 822.6 m above sea level. Plots were split into elevational bands in line with previous investigations of elevational thresholds between lowland and montane forests in Hong Kong (Zhuang & Corlett, 1997). While lowland forests are typically found below 500 m, Montane forest in Hong Kong in the Tai Mo Shan and Tai Po Kau area occur at elevations above 500 m, and high plots were thus placed above the 500 m elevational boundary for the following analysis (Corlett, 2011; Zhuang & Corlett, 1997). Low‐ and mid‐elevation bands were split to represent equal numbers of age replicates below the montane forest level, which would facilitate fine‐grained analysis of community compositional responses to increasing elevation. Woody species were surveyed across the plots and species presence and abundance data was recorded.

### 2.1 Beta diversity calculations

Analysis followed the pairwise beta diversity framework developed by Baselga (2010) to quantify species richness dissimilarly between paired plots. The framework calculates dissimilarity metrics between all paired plots and partitions total beta diversity dissimilarity into additive subcomponents of turnover (Simpsons diversity) and nestedness, which describe species replacement and species loss/gain, respectively. The turnover component is mathematically identical to the Simpson dissimilarity index and total beta diversity is equivalent to the Sorensen dissimilarity index. Since total beta diversity is the sum of species turnover and nestedness, the nestedness component is calculated as the difference between the Sorensen dissimilarity index and the Simpson dissimilarity index. Sorensen pairwise dissimilarity (*β*_sor_), and the subcomponents of Simpson pairwise dissimilarity (*β*_sim_) and nestedness‐resultant dissimilarity (*β*_sne_) as indices of beta diversity were generated using the R package betapart as pairwise dissimilarity matrices (Baselga & Orme, 2012). Mean turnover and mean nestedness values were calculated to assess the relative contribution of each beta diversity subcomponent to overall beta diversity patterns.

### 2.2 Generalized dissimilarity modelling and comparisons of beta diversity subcomponents

In order to account for curvilinearity of rates of turnover and nestedness along environmental gradients, generalized dissimilarity modelling (GDM) was selected for construction of models of beta diversity subcomponents in response to temporal and environmental variable matrices (Ferrier et al., 2007). GDM models biological variation as a function of environment and geography using distance matrices, by relating biological dissimilarity between sites to the extent to which sites differ in their environmental conditions (environmental distance) and how isolated they are from one another (geographical distance). To address the questions of the present study, turnover and nestedness were modelled as functions of plot age (years), plot elevation (m), inter‐plot distance (m), in addition to soil variables of organic matter content (g kg^−1^) and soil moisture saturation (%). Preliminary testing with additional soil and topographical indices indicated no affect on turnover and nestedness and these variables were subsequently discarded from onward analyses. All GDM models were fitted using the R package gdm. Models were fitted with default number of three i‐spline basis functions, while knots were set at default values of 0 (minimum), 50 (median), and 100 (maximum) quantiles. Summary statistics of percent deviance explained, intercept, fitted i‐splines per temporal/environmental predictor included, knot coefficients and sums, were generated. Sums determined the amount of compositional turnover or nestedness with all other variables constant. Plots were generated for i‐splines of each temporal and environmental predictor, in addition to plots of (i) fitted relationships between predicted ecological distance and observed compositional dissimilarity and (ii) predicted versus observed biological distance. Plots were broken down into elevational bands in line with the aforementioned zonation of Hong Kong secondary forest types between lowland and montane at the 500 m.a.s.l threshold, and analyses re‐ran with simplified models to determine the contributions of age and distance at different elevations (Zhuang & Corlett, 1997). While lowland forests are typically found below 500 m, montane forest in Hong Kong in the Tai Mo Shan and Tai Po Kau area were previously found to occur at elevations above 500 m, and high plots were thus placed above the 500 m elevational mark (Corlett, 2011; Zhuang & Corlett, 1997). Low‐ (200 m–300 m) and mid‐elevation (300 m < 500 m) bands were split to represent equal numbers of age replicates below the montane forest threshold, and facilitate fine‐grained analysis of community compositional responses to increasing elevation. ANOVA and Kruskal Wallis testing were used to determine variation within and between elevational bands in turnover and nestedness. Sites were pooled within elevational bands and patterns of turnover and nestedness were assessed.

### 2.3 Non‐metric multidimensional scaling

To assess associations of plant species in multidimensional space and identify species assemblages associated with successional age classes, non‐metric dimensional scaling was conducted using the R package vegan. The model was built from plot plant species abundance and environmental datasets. NMDS analyses were built upon a Bray–Curtis dissimilarity matrix of all environmental variables measured. ANOSIM analysis was carried out with 9999 permutations to assess community dissimilarity between age classes.

### 2.4 Assessment of species richness & functional group

Surveyed plant species were categorized into plant functional groups including canopy tree, small tree, shrub, and scandent shrub or liana, and percentage cover statistics were generated and compared between age classes. Species richness and Shannon's diversity metrics were generated in the R package vegan, and one‐way ANOVA was used to test differences in species richness and diversity between age classes.

## 3 RESULTS

### 3.1 Turnover & nestedness generalized dissimilarity modelling

**FIGURE 2:**
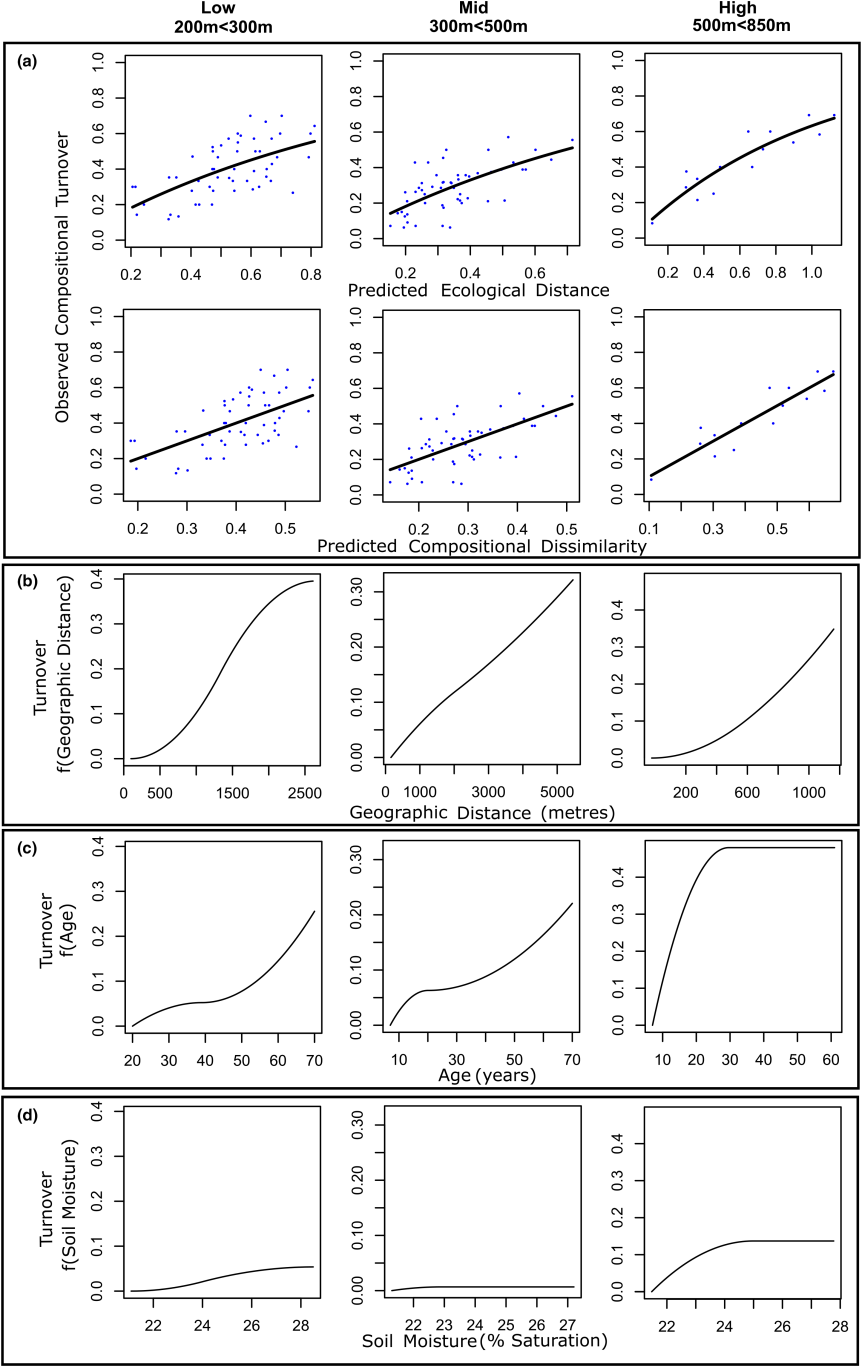
GDM models of turnover displaying (a) fitted relationships between predicted ecological distance (summed transformed pairwise environmental dissimilarities of study sites) and observed compositional dissimilarity (pairwise Simpson's beta diversity) and predicted versus observed biological distance and; I‐spline function plots of plant species community turnover explained by transformed predictor variables including (b) geographic distance between communities (metres); (c) age dissimilarity between communities (years) and; (d) Soil Moisture Saturation (%) dissimilarity between communities.

**FIGURE 3:**
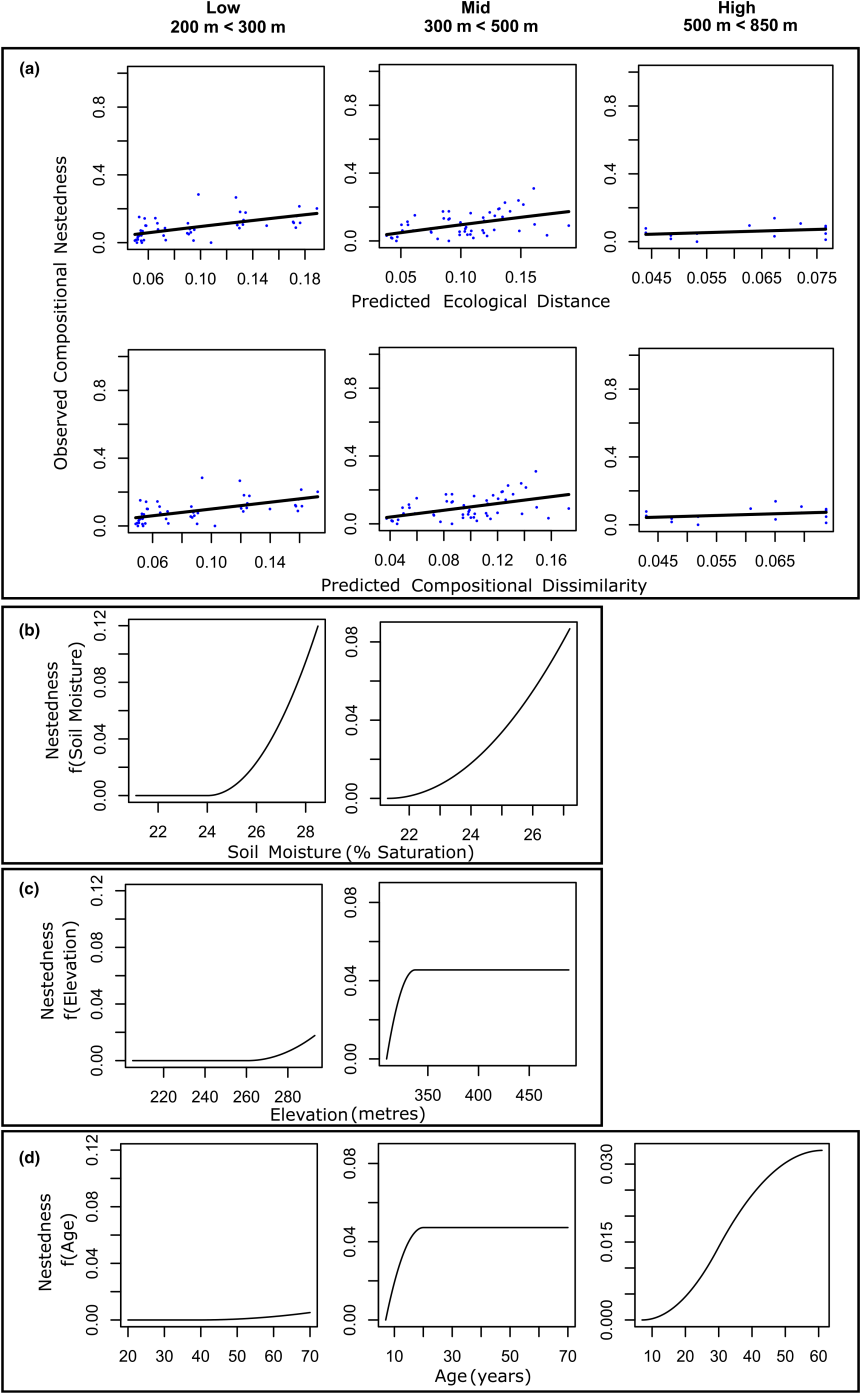
GDM models of Nestedness displaying (a) fitted relationships between predicted ecological distance (transformed pairwise environmental dissimilarities of study sites) and observed compositional dissimilarity (pairwise nestedness beta diversity) and predicted versus observed biological distance and I‐spline function plots of plant species community nestedness against transformed predictor variables including (b) soil moisture saturation (%) dissimilarity between communities; (c) Elevational dissimilarity between communities within elevational bands and; (d) Age dissimilarity (years) between communities.

Generalized dissimilarity modelling was conducted upon input matrices of Simpson's beta diversity (species turnover) and species nestedness at low, mid, and high elevational bands. Models of turnover explained 36.4 of deviation at low elevations (Table 1). Inter‐community distance was the primary predictor of species turnover at low elevations (Sum of Coefficients: 0.395), followed by plot age (Sum of Coefficients: 0.259) and soil moisture (Sum of Coefficients: 0.054) (Table 1). Turnover increased with increasing geographic distance and age dissimilarity, and the rate of turnover increased with increasingly large dissimilarities in geographic distance and age between plots. Models of turnover explained 30.751% of deviation at middle elevations, with inter‐community distance as the primary explanatory variable of deviation (Sum of coefficients: 0.324), followed by age (Sum of coefficients: 0.223), elevation (Sum of coefficients: 0.19), and soil moisture (0.007) (Table 1). Similarly to low‐elevation plots, middle‐elevation plant communities exhibited increasing rates of turnover with increasing dissimilarities in inter‐community distance, age and soil moisture, while within‐band elevational discrepancies were also influential upon rates of turnover. Models of turnover explained 83.45% of deviation at high elevations, with age acting as the primary explanatory variable of deviance (Sum of coefficients: 0.48), followed by soil moisture (Sum of coefficients: 0.352), elevation (Sum of coefficients: 0.172), and geographic distance (Sum of coefficients: 0.137) (Table 1). Turnover increased sharply with increasing geographic distance at all elevational bands (Figure 2b), while the turnover exhibited a higher rate with higher levels of dissimilarity in age at low and middle elevations, with a sharper rise and plateau in rate of turnover with age dissimilarity at higher elevations (Figure 2c). Turnover exhibited lower rates in response to increasing dissimilarities in soil moisture (Figure 2d). Models of nestedness at low elevational bands explained 32.468% of overall deviance (Table 1). Soil moisture was the primary explanatory variable (Sum of coefficients: 0.122), followed by Elevation (Sum of coefficients: 0.018) and age (Sum of coefficients: 0.005). At mid‐elevations, modelling explained 22.837% of deviance, with soil moisture as the primary explanatory variable (Sum of coefficients: 0.088), followed by age (Sum of coefficients: 0.047), and elevation (Sum of coefficients: 0.045) (Table 1). Models of nestedness at high elevations explained 8.515% of deviance, with age as the sole explanatory variable (0.033) (Table 1). Nestedness exhibited a higher rate with higher levels of soil moisture dissimilarity at low and middle elevations while having no influence upon models of nestedness at high elevations (Figure 3b). Nestedness exhibited higher rates only with higher levels of elevational dissimilarity within the low elevational band, while in the middle elevational band nestedness exhibited a sharp rate before plateauing with increasing elevational dissimilarity (Figure 3c). Within the low elevational band, nestedness increased at a low rate with increasing age dissimilarity, while in the middle elevational band community nestedness increased with a high rate with small differences in age, before plateauing (Figure 3d). In the high elevational band, community nestedness increased in a sigmoidal curve with increasing age dissimilarity (Figure 3d).

**TABLE 1:** Summary of generalized dissimilarity model (GDM) with significant variables presented. Relative importance of variables is determined by summing I‐spline coefficients from GDMs.

	Null deviance	GDM deviance	%deviance explained	Variables	Coefficients
**Low**					
*β*_Sim_	5.45	3.466	36.405	Geographic Distance	0.395
				Age	0.259
				Soil Moisture	0.054
*β*_Sne_	2.919	1.92	32.468	Soil Moisture	0.122
				Elevation	0.018
				Age	0.005
**Mid**					
*β*_Sim_	4.51	2.62	39.751	Geographic Distance	0.324
				Age	0.223
				Elevation	0.19
				Soil Moisture	0.007
*β*_Sne_	2.995	2.311	22.837	Soil Moisture	0.088
				Age	0.047
				Elevation	0.045
**High**					
*β*_Sim_	2.044	0.338	83.45	Age	0.48
				Soil Moisture	0.352
				Elevation	0.172
				Geographic Distance	0.137
*β*_Sne_	0.431	0.394	8.515	Age	0.033

Turnover contributed relatively higher proportions to beta diversity at all elevational bands, with turnover representing 81.918% of Sorensen beta diversity in the low elevational band, 75.045% in the middle elevational band and 87.807% in the high elevational band (Table 2). Nestedness contributed a smaller proportion to overall Sorensen beta diversity at all elevations, representing 18.028% of Sorensen beta diversity in the low elevational band, 24.955% in the middle elevational band and 12.193% in the high elevational band (Table 2). ANOVA and Kruskal Wallis testing found that rates of turnover and nestedness did not significantly differ between elevational bands or age groups (*p* > .05). Rates of turnover (*β*_Sim_: 0.069767) and nestedness (*β*_Sne_: 0.010692) were lower between 200 and 300 m and 300–500 elevational bands than between these bands and 500–850 m communities (*β*_Sim_: 0.172414; *β*_Sne_: 0.170052) (Table 3).

**TABLE 2:** Total *β*‐diversity (Sorenson *β*_Sor_), Simpson dissimilarity (species turnover *β*_Sim_), and nestedness (*β*_Nes_), for plant communities at low, mid and high elevational bands, with percentages of mean Sorensen beta diversity (% mean).

Band	Component	Minimum	1st Qu	Median	Mean	3rd Qu	Maximum	% mean
200–300 m	*β*_Sim_	0.118	0.300	0.389	0.402	0.512	0.700	81.918
	*β*_Sne_	0.000	0.042	0.080	0.089	0.117	0.284	18.082
	*β*_Sor_	0.189	0.380	0.486	0.491	0.600	0.806	100
300–500 m	*β*_Sim_	0.063	0.205	0.286	0.287	0.372	0.571	75.045
	*β*_Sne_	0.000	0.043	0.075	0.095	0.140	0.310	24.955
	*β*_Sor_	0.156	0.294	0.368	0.382	0.457	0.636	100
500–850 m	*β*_Sim_	0.083	0.310	0.400	0.437	0.592	0.692	87.807
	*β*_Sne_	0.000	0.034	0.052	0.061	0.088	0.138	12.193
	*β*_Sor_	0.154	0.366	0.538	0.497	0.618	0.724	100

**TABLE 3:** Pairwise turnover (*β*_Sim_) and nestedness (*β*_Sne_) indices of plant species between elevational bands based on species presence and absence for each of the three elevational bands.

Elevation (m)	200–300 m	300–500 m	500–850 m
***β***_**Sim**_			
200–300 m	0		
300–500 m	0.069767	0	
500–850 m	0.172414	0.172414	0
***β***_**Sne**_			
200–300 m	0		
300–500 m	0.010692	0	
500–850 m	0.170052	0.16092	0

Turnover contributed higher proportions to overall Sorenson beta diversity than nestedness within all successional age groups, with turnover representing 82.64% of Sorensen beta diversity in the 7‐year age group, 83.002% in the 20‐year age group, 79.483% in the 39‐year age group, 91.641% in the 61‐year age group, and 68.872% in the 70‐year age group (Table 4). Nestedness contributed a smaller proportion to overall Sorensen beta diversity within all successional age groups, representing 17.326% of Sorensen beta diversity in the 7‐year age group, 16.998% in the 20‐year age group, 20.517% in the 39 year age group, 8.359% in the 61 year age group, and 31.128% in the 70 year age group (Table 4). Rates of turnover were high between plots in the 61‐ and 70‐year age groups and younger age groups (Table 5), such as between age groups 7 and 70 (*β*_Sim_: 0.413793) and also between age groups 7 and 61 (*β*_Sim_: 0.1). Nestedness showed an inconsistent pattern between different age groups, with values >0.1 between age group seven and age groups 20, 39 and 61 (Table 5), while in other instances no nestedness was observed, such as between age groups 20 and 61 (Table 5).

**TABLE 4:** Total *β*‐diversity (Sorenson *β*_Sor_), Simpson dissimilarity (species turnover *β*_Sim_), and nestedness (*β*_Nes_), for plant communities in 7‐,20‐, 39‐, 61‐ and 70‐year‐old communities, with percentages of mean Sorensen beta diversity (% mean).

Age (years)	Component	Minimum	Qu 1	Median	Mean	Qu 3	Maximum	% mean
7	*β*_Sim_	0.071	0.364	0.400	0.416	0.421	0.800	82.674
	*β*_Sne_	0.000	0.025	0.089	0.087	0.139	0.200	17.326
	*β*_Sor_	0.235	0.435	0.474	0.504	0.575	0.833	100.000
20	*β*_Sim_	0.136	0.254	0.400	0.407	0.500	0.833	83.002
	*β*_Sne_	0.017	0.035	0.078	0.083	0.118	0.183	16.998
	*β*_Sor_	0.156	0.360	0.455	0.491	0.606	0.852	100.000
39	*β*_Sim_	0.125	0.250	0.323	0.370	0.445	0.733	79.483
	*β*_Sne_	0.000	0.051	0.099	0.096	0.124	0.239	20.517
	*β*_Sor_	0.243	0.347	0.458	0.466	0.562	0.742	100.000
61	*β*_Sim_	0.118	0.255	0.629	0.517	0.741	0.786	91.641
	*β*_Sne_	0.008	0.019	0.051	0.047	0.061	0.113	8.359
	*β*_Sor_	0.231	0.318	0.681	0.564	0.763	0.806	100.000
70	*β*_Sim_	0.143	0.225	0.300	0.311	0.391	0.500	68.872
	*β*_Sne_	0.015	0.086	0.134	0.140	0.201	0.267	31.128
	*β*_Sor_	0.294	0.421	0.451	0.451	0.505	0.576	100.000

**TABLE 5:** Pairwise turnover (*β*_Sim_) and nestedness (*β*_Sne_) indices of plant species between elevational bands based on species presence and absence 7‐, 20‐, 39‐, 61‐ and 70‐year‐old communities.

Age (years)	7	20	39	61	70
***β***_**Sim**_					
7	0				
20	0.066667	0			
39	0.033333	0.026316	0		
61	0.1	0.131579	0.078947	0	
70	0.413793	0.206897	0.068966	0.206897	0
***β***_**Sne**_					
7	0				
20	0.109804	0			
39	0.193333	0.082118	0		
61	0.105882	0	0.077679	0	
70	0.009936	0.106536	0.201305	0.106536	0

### 3.2 Non‐metric dimensional scaling

**FIGURE 4:**
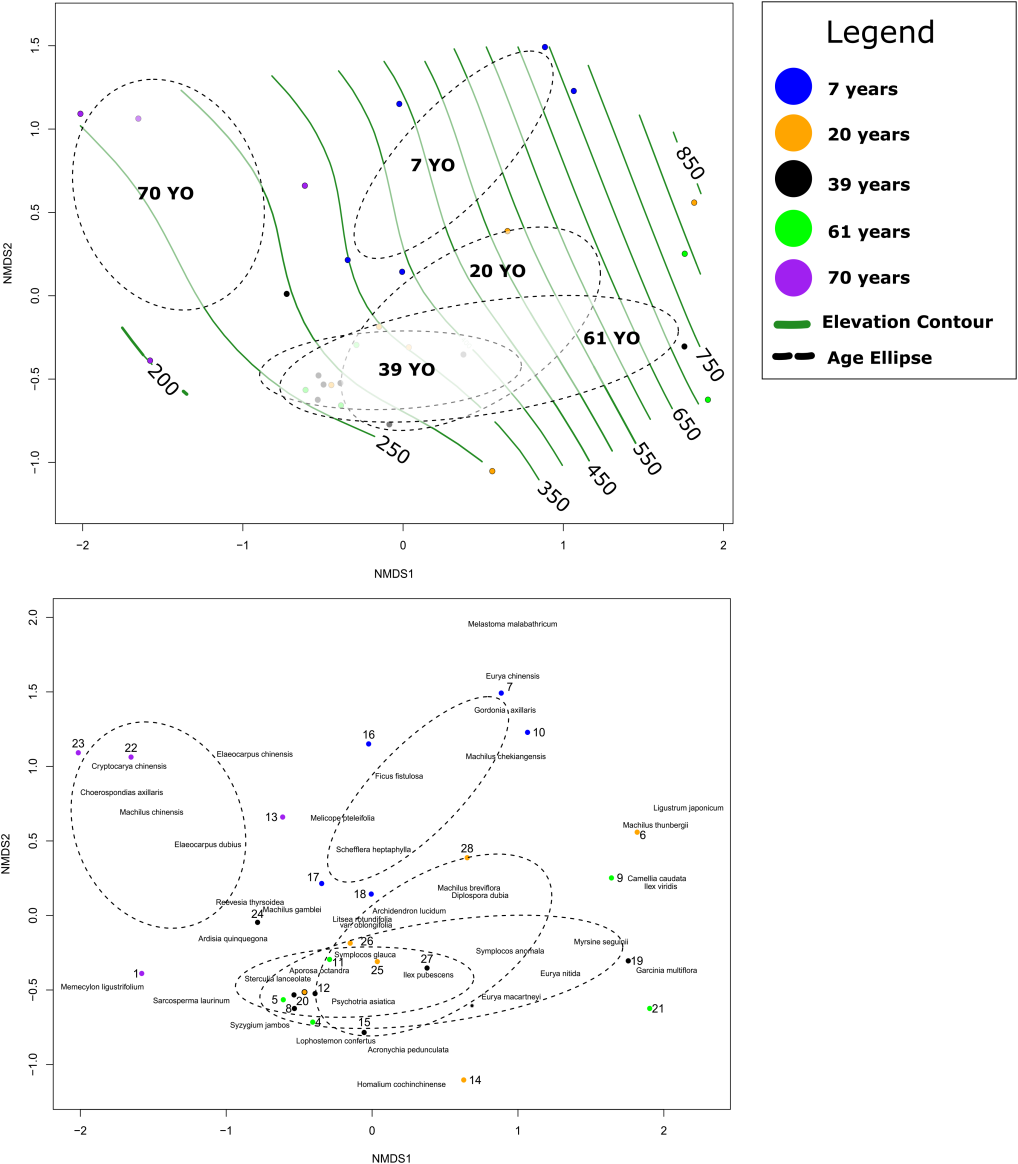
Non‐Metric Dimensional Scaling Analysis (*R* ^2^ = 0.915) with ellipses designating 7‐year plots (blue), 20‐year plots (orange), 39‐year plots (black), 61‐year plots (green), and 70+ year plots (purple), with additional elevational contours in green and species (grey).

NMDS analysis with a well‐fitted model (*R* ^2^ = 0.915) exhibited clustering of the middle‐age plot categories in species composition and environmental parameters in multidimensional space (Figure 4). ANOSIM found that community composition significantly differed with age class (*p* < .05). The forest age categories of 20, 39 and 61 years since succession from open grassland showed a clustering of some shared species. *Diplospora duplia*, *Machilus* spp., *Symplocos* spp., *Litsea rotundifolia var. oblongifolia*, *Ilex pubescens*, and *Psychotria asiatica* comprised the group of species showing heightened overlap between middle‐aged forest classes (Figure 4). The 7 year and 70+ year age classes showed distinct assemblages relative to the middle‐age plots, with little to no overlap in representation. In the 7‐year age class, *Ficus fistulosa*, *Melicope pteleifolia*, and *Ilex asprella* were prevalent (Figure 4). In the 70+ age class, *Elaeocarpus dubius*, *Machilus chinensis* and *Cryptocarya chinensis* were more common (Figure 4).

### 3.3 Species richness & functional groups

8575 woody individuals of DBH ≥ 1 were measured, and their species were identified. They belong to 229 species and 63 families. Two species from *Symplocos*, *Machilus* were identified at genus level, and one species from *Lauraceae* was identified at family level. Three gymnosperm species were recorded, including *Cunninghamia lanceolata*, *Gnetum luofuense* and *Pinus elliottii*. *Acacia confusa*, *Duranta erecta*, *Eucalyptus exserta*, *Lantana camara*, *Lophostemon confertus* and *Pinus elliottii* which are exotic, were recorded. *Cunninghamia lanceolata* and *Dimocarpus longan* are species native to China and historically cultivated in the region. The number of species recorded increased sharply in the first 20 years of succession. It reached a steady rate following 20 years and dropped after 70 years. One‐way ANOVA showed non‐significant difference in species richness and Shannon's species diversity between different forest age classes (*F* (1, 26) = 0.535, *p* = .471). One‐way ANOVA also showed no variation between density of individuals and between age classes. However, the median density in 7‐year‐old forests was 170/400 m^2^ (4250/ha), while it ranged from 201 to 328/400 m^2^ (8200/ha) for other ages. Tree functional groups were divided into canopy tree, shrub and scandent shrubs or lianas. The proportion of canopy tree species rose from 24.5% in the first 20 years to 50% in a mature forest. At the same time, the number of shrub species decreased from 41.83% to 20.6% with the peak in the mid‐age forest (20–61 years), as well as scandent shrubs or lianas (3.1% in 61 years old). The number of small trees did not change drastically across age classes (19.3% to 26.5%).

## 4 DISCUSSION

The study of secondary forest succession is essential for determining regenerative pathways and conservation priorities for degraded landscapes, and elevational and spatial gradients within regenerating forests may reflect differentiation between upland and lowland ecosystems and barriers to dispersal (Nichol & Abbas, 2021). In the present study, secondary forest regeneration was assessed along successional, topographic, spatial and environmental gradients to determine community assembly mechanisms via responses of plant species turnover and nestedness, as well as the change in species composition and proportion of plant functional groups across the forest successional continuum. We found that as forests became more dissimilar in age, species turnover increased, and a shift in functional groups from shrub dominance to tree dominance occurred between early and late successional stages. We found that the youngest (7 year) and oldest (70 year) patches had distinct communities, while communities aged between 20 to 61 years showed compositional overlap. Community assembly mechanisms were differentiated between lowland and montane forest type, with intercommunity distance as the most influential driver of turnover in the former and forest age as the most influential driver of turnover in the latter, although forest age was influential across elevations. High rates of turnover occurred between lowland and montane forest plant communities, suggesting differential community assembly in lowland and montane forests in Hong Kong.

### 4.1 Species turnover and functional differentiation during succession

Across lowland and montane forest, plant species exhibited increasing turnover with increasing age dissimilarity between communities as hypothesized (H1), although this was secondary to the importance of inter‐community distance in lowland forest. Additionally, we found that a gradual shift in the proportion of shrub versus tree species occurred with the transition from early successional communities with a higher prevalence of shrub species, to later successional communities dominated by tree species. As previously noted, there is a break between Lowland and Montane plant community species composition in Hong Kong, and our finding that communities exhibited differential community assembly mechanisms between these forest types may be expected due to the low winter temperatures experienced at high elevations filtering out lowland specialists (Corlett, 1999; Dudgeon & Corlett, 1994; Zhuang & Corlett, 1997). Irrespective of this, turnover can be impacted by competition, dispersal filtering and environmental filtering, and the increase in turnover through successional time in lowland and montane forest may indicate deterministic environmental filtering and competitive mechanisms shaping plant community assembly during secondary succession in Hong Kong subtropical forests (Baselga, 2010; Conradi et al., 2017). Significantly, turnover of species and functional groups with increasing forest age may reflect structural, abiotic and biotic shifts during secondary succession (Chai et al., 2019; Perring et al., 2018). In classical descriptions of secondary succession, open environments such as post‐fire grasslands in Hong Kong with harsh light, thermal and soil conditions are colonized by fast‐growing early successional pioneer grass and shrub species, before gradually transitioning with the arrival of slow‐growing mid‐to‐late‐successional small‐ and canopy‐tree species (Horn, 1974; Odum, 1969; Perring et al., 2018). These shifts in composition are thought to cause forests to undergo microclimatic shifts, resulting in a cooler, shadier and moister understorey, a closed canopy, and increased competition for light and nutrient resources. These abiotic and biotic changes may facilitate a transition from community assembly where environmental filtering is the primary filter of species, to community assembly where interspecific competition for limited light and nutritive resources comes to dominate, as well as a transition from fast‐growing to slow‐growing plant species (Lohbeck et al., 2014).

In the present study, the youngest communities in the regenerating subtropical forests exhibited a mixture of shrubs (*Melicope pteleifolia*, *Eurya chinensis*) and fast‐growing small trees (*Ficus fistulosa*, *Schefflera heptaphylla*), which would be expected in taller shrublands starting to be encroached upon by mid‐successional species such as *Machillus* spp. (Dudgeon & Corlett, 2011; Hendrayana et al., 2021). Middle‐aged community membership exhibited overlapping shrub and tree species which would be expected within shrublands and those in forest patches with a developing canopy. Shrub species such as *Psychotria asiatica*, which is prominent throughout the understorey of secondary forests in Hong Kong, and *Ilex pubscens*, were prevalent across 20‐ to 61‐year‐old communities alongside several tree species, including *Machillus* spp., and species showing shrub and small tree intermediary growth forms such as lowland species *Symplocos glauca*, and species common on slopes and in ravines such as *Eurya macartneyi*. This mixture of understorey shrubs, taller shrubs and small trees alongside some trees capable of growing to heights of 20 m in middle‐aged communities may indicate that compositionally and functionally, the transition from early successional phases dominated by shrubs to later successional communities dominated by large trees requires time frames of 20 to 60 years, which is more in line with the higher estimates of time required for secondary succession (Bowen et al., 2007; Poorter et al., 2021). The oldest communities were dominated by trees, such as *Eleocarpus dubius*, *Machillus chinensis*, and *Cryptocarya chinensis* among others, which is in line with expectations for forest plant communities 70 years of age since the onset of succession at the time of surveying (Zhu et al., 2023). Previous investigations suggest that these older communities do not show consistent convergence, which may challenge the notion of the appearance of a ‘climax’ community with increasing age of forests, and support the notion of a dynamic mosaic across successional landscapes (Abbas et al., 2019; Horn, 1974). Another consideration is the underrepresentation of Fagaceae genera in these older communities, which face significant dispersal barriers in Hong Kong secondary forests (Dudgeon & Corlett, 2011). Subsequently, our finding that species turnover and prevalence of trees increased while shrub species decreased with increasing dissimilarities in community age may indicate turnover of early to mid to late successional species along the forest regenerative continuum, which implicates abiotic and biotic changes during secondary succession.

Tropical forests undergoing secondary succession in Mexico saw temperature decreases of up to 75% of open sky radiation and humidity increases of up to 74% in later succession relative to the start of early succession (Lebrija‐Trejos et al., 2011). The role of age in regenerating subtropical forests in Hong Kong may reflect similar abiotic changes in selecting for species assemblages during the process of secondary succession which are adapted to the altered environment of older forests as a canopy develops (Muscarella et al., 2016; Zobel et al., 1993). This may also support the hypothesized increase in biotic filtering from competition for light resources, which has been found in functional and niche responses to succession in Puerto‐Rican tropical forests (Muscarella et al., 2016). Our finding that tree functional groups shifted from a high extent of shrub species to dominance of tree canopy species along the successional gradient, and that soil moisture was also influential upon species turnover suggests a shift in competitive regimes in regenerating subtropical forests in Hong Kong. Tree species turnover increases have been found to occur with successional age in tropical forests in Venezuela undergoing secondary succession across smaller chrono‐sequences of 0–20 years compared to the 7‐ to 70‐year continuum in the present study (Villa et al., 2021). Conversely, an investigation of tropical forest succession in Mexico along a successional gradient of 0–60 years found that total beta diversity declined with increasing successional age (López‐Martínez, Hernández‐Stefanoni, et al., 2013; López‐Martínez, Sanaphre‐Villanueva, et al., 2013). These studies were conducted without accounting for elevation, or over narrow elevational gradients (60–160 m) relative to the broader elevational gradient in our study (200–850 m), while meta‐analyses and studies elsewhere in subtropical southern China did not integrate elevation and inter‐community distance as predictors of community compositional dissimilarity during secondary succession. Correcting for elevational and spatial effects upon community assembly with increasing successional age is necessary, as elevational gradients significantly alter abiotic conditions (Willig et al., 2011). In the present study, subtropical lowland and montane forest plant communities in Hong Kong exhibited increasing species and functional turnover with increasing forest age, while the influence of spatial variables in lowland forests implicates additional mechanisms leading to the formation of plant communities at lower elevations.

### 4.2 Spatial turnover in lowland forest

Our analysis found that within lowland forest types, geographic distance was the primary driver of plant species turnover (H2). Several factors are known to drive turnover, although dispersal limitations are generally held to limit turnover at larger geographical scales in South China than those in the present study (Chen et al., 2016). Investigations in Ding Hu Shan, Guangdong, China, previously found that shrubs had a greater capacity for dispersal than tree species (Bin et al., 2010). Our finding that turnover is higher within rather than between age and elevational categories and the significance of inter‐community distance in lowland forests suggests a high degree of spatial plant species turnover and subsequently dispersal limitation of plant species in secondary subtropical lowland forests in Hong Kong.

These trends observed in regenerating forests may be driven by regional and idiosyncratic regional dispersal constraints, as well as complex land use histories and soil quality heterogeneity. Previous investigations of the distribution of flora in Hong Kong, the low plant diversity in frugivore droppings and seed traps, the absence of native scatter‐hoarding rodents and low diversity of large‐gaped bird species for the dispersal of larger fruits have led to the suggestion that dispersal networks in the region are degraded (Corlett, 2011). However, it is also notable that many seed and fruit‐dispersing species have returned to Hong Kong in the early 21st century, such as Greater Necklaced Laughingthrush and Pallas squirrel (Dudgeon & Corlett, 2011; Leven & Corlett, 2004). Notwithstanding, tree species in the family Fagaceae (oaks, chestnuts) and genera in other families such as Camellia and Styrax have large dry fruits with no outer fleshy layer, which are typically dispersed by scatter‐hoarding rodents and specialist birds. Species within these groups are thought to be dispersal limited in Hong Kong, and yet they do not feature prominently in the communities in the present study. A more general pattern of dispersal limitation may be observed in these data, which requires further research into the distribution of dispersal syndromes and seed and fruit sizes to verify. Three species of birds including *Pycnonotus sinensis*, *Pycnonotus jocosus* and *Zosterops japonicus* account for over two‐thirds of seed dispersal in Hong Kong, with *Pycnonotus* particularly important for forest seed dispersal between patches (Corlett, 2011). The seed dispersal range of *P. sinensis* has been previously found to be between 100 and 1000 m, while the seed rain created by the three aforementioned bird species is dominated by smaller seeds which are <14 mm in diameter. Rates of turnover across spatial gradients did not saturate in communities in Hong Kong, but were steep and increased over this dispersal range. Spatial patterns of turnover may reflect dispersal ranges of major seed‐dispersing avifauna. Bat species in Hong Kong, which strongly favour *Ficus* spp., have been found to disperse seeds to a maximum distance of 150 m (Corlett, 2011), which may further contribute to the observations in the presented work.

Another potential explanation of the patterns of spatial turnover we observed is the lack of adjacent primary forests in Hong Kong. A study on a matrix of primary tropical and secondary forests in Mexico found that frugivore dispersers were more likely to carry late‐successional seeds to secondary forest patches that were closer to primary tropical forests (Vleut et al., 2015). Owing to historical deforestation and a lack of nearby old‐growth forest, there are fewer opportunities for dispersal by frugivore species in Hong Kong. However, the relationship between turnover and dispersal has been contested and inconsistently been found to be driven by spatial variables alone (Chen et al., 2016) and without further investigation of dispersal syndromes and surveys of recent frugivore dietary compositions, strong conclusions remain evasive (Soininen et al., 2018). Functional analysis of dispersal syndromes of indicator species at different successional stages may clarify whether degraded dispersal mechanisms are responsible for the observed patterns of turnover in the present study. Irrespective of these potential future investigations, it is clear that community assembly responds differentially between lowland and montane subtropical forest in Hong Kong, and the primacy of spatial variables at lower elevations may implicate prolonged dispersal limitations in the region. The closest analogous forest type to old growth or primary forest in Hong Kong are Feng Shui forests, some of which are thought to be up to 400 years old, such as a woodland growing in the Shing Mun Country Park (Dudgeon & Corlett, 2011). However, the capacity for plant species to disperse to secondary forest has been drawn into question by previous investigations of plant distribution, and their composition is not thought to resemble that of the primary evergreen tropical forests which once grew in Hong Kong prior to the onset of agricultural development and land clearance (Zhuang & Corlett, 1997).

### 4.3 Differentiation between lowland and montane forest community assembly

Elevational gradients integrate a breadth of abiotic changes which may serve to support a deterministic view of community assembly within the context of secondary succession in the tropical forests of Hong Kong (Muscarella et al., 2020). We found a higher degree of turnover between lowland and montane forest as hypothesized (H3), which may reflect the differential selective regimes presented with large shifts in community elevation. We found that *Machilus breviflora* tended to be present within Montane forest, in addition to *Myrisine* spp., *Eurya* spp., *Ilex* spp., *and Camellia* spp., while Lowland communities tended to exhibit *Psychotria* spp. and *Schefflera* spp., which is consistent with previous zonations of Hong Kong flora by forest type (Zhuang & Corlett, 1997). Forest communities are exposed to low winter temperatures below 10°C in Hong Kong at higher elevations above 500 m, and plant species such as *P. asiatica*, *Acronychia pedunculuta*, *Aporosa dioica* and *Garcinia oblongifolia* are usually absent at these high elevations (Dudgeon & Corlett, 2011). Temperatures below 10°C can cause chill damage to tropical plants, and Hong Kong is notable for having a unique flora comprising a mixture of lowland tropical plant families, in addition to plant families with typically non‐tropical distributions. Clines in temperature and moisture along elevational gradients can drive significant changes in abiotic conditions, altering the selective regimes that communities are subjected to, and this may explain the pattern of differential community assembly between lowland and montane forests in Hong Kong (Descombes et al., 2017).

This is consistent with other investigations within tropical contexts, such as work by (Swenson et al., 2011), which found that turnover of tree species and functional diversity was significantly predicted by elevation along an elevational gradient in tropical forests in Puerto Rico. However, while this study took place over a broader elevational gradient (100–1075 m) than the present study, it did not assess interactive changes along successional trajectories or the effects of inter‐community distance, which were found to nuance patterns of plant species turnover in the present study. Research investigating responses of tropical dry forests to succession and elevation simultaneously found that the influence of age, elevation and distance upon beta diversity was functional‐group dependent (López‐Martínez, Hernández‐Stefanoni, et al., 2013; López‐Martínez, Sanaphre‐Villanueva, et al., 2013). This phenomenon has been found to occur in multiple plant functional groups during succession in temperate forests, where both elevation and successional age are key in driving species responses (Hilmers et al., 2018). Assessment of plant functional diversity responses to successional age and elevation in our study system may help to clarify plant functional group‐specific effects of these variables in driving successional trajectories in Hong Kong sub‐tropical forests.

### 4.4 Nestedness, soil moisture & succession

Nestedness typically represents a minor subcomponent of overall Sorenson beta diversity compared to turnover in additive models, and responses of this metric to age, elevation and inter‐community distance may reveal patterns of species loss or gain, species richness differences, or extinction‐colonization dynamics (Baselga, 2010; Si et al., 2016). We found that across elevational and age groups, nestedness contributed a smaller proportion of overall Sorensen beta diversity, with marked increases in line with increasing dissimilarity in soil moisture, age and within‐band elevation. The finding that nestedness increased with increased age dissimilarities between communities did not align with other studies such as Villa et al. (2021) wherein species nestedness was found to be lower in older plots. However, the narrower successional gradient used in their study (0–20 years) limits comparisons to our findings. Notwithstanding, Villa et al. (2021) found that soil properties played a more significant role in shaping patterns of nestedness during secondary succession, which are concordant with our own findings. Although we found no correlation of soil moisture with age or elevation, it can be speculated that soil moisture may reflect alterations to the abiotic selective regime apparent within each community, such as the degree of canopy closure. Temperature and canopy cover and structure readings are not available, and such measurements could clarify the role of soil moisture further in predicting nestedness of plant communities in the present study. In non‐tropical systems, investigations in Mediterranean grassland succession found that nestedness also increased with age (Bagaria et al., 2019), while studies in grassland communities found that communities with poor dispersal mechanisms exhibited higher levels of nestedness overall (Conradi et al., 2017). Integrating remote sensing and functional trait metrics may help to delineate the drivers of patterns of nestedness as well as turnover in the regenerating subtropical forests of Hong Kong.

## 5 CONCLUSION

The present study found that inter‐community distance and age played a significant role in driving observations in species turnover and nestedness, while the latter was also strongly influenced by soil properties, and clear variation was observed between Lowland and Montane forest types. The finding that inter‐community distance was the primary driver of plant species turnover in lowland forests suggests a continued issue of dispersal limitations in Hong Kong's secondary forests and necessitates consideration of conservation measures. Specifically, the reintroduction of key mammalian dispersing fauna with scatter‐hoarding behaviours such as Edwards Long Tailed Rats, and the active planting of late‐successional Fagaceae tree species in taller shrublands may facilitate the resumption of dispersal where dispersal networks are still recovering or not functionally robust. It is also clear that a functional assessment of plant dispersal trait distributions may permit a deeper understanding of the distances that tree species are able to disperse from individual forest patches. Notwithstanding, a clear shift in community composition was apparent between successional groups with increasing community age. The finding that turnover dominated overall Sorensen beta diversity has significant implications for conservation and supports the continued protection of a large number of sites due to the high degree of spatial variability in species composition.

## AUTHOR CONTRIBUTIONS

**Coskun Guclu:** Conceptualization (lead); formal analysis (lead); investigation (lead); methodology (lead); writing – original draft (lead); writing – review and editing (lead). **Chung‐Lim Luk:** Conceptualization (lead); formal analysis (lead); investigation (lead); methodology (lead); writing – original draft (lead); writing – review and editing (lead). **Louise Amy Ashton:** Supervision (lead); writing – review and editing (lead). **Sawaid Abbas:** Data curation (lead); investigation (lead); resources (lead). **Michael J. W. Boyle:** Investigation (lead); methodology (lead); supervision (lead); writing – original draft (supporting); writing – review and editing (supporting).

## FUNDING INFORMATION

MJWB was funded by the National Natural Science Foundation of China Excellent Young Scientist Award (AR215206), and the University of Hong Kong 46th Round Fellowship. CG was funded by the Hong Kong University Grants Council Hong Kong PhD Fellowship and the University of Hong Kong Presidential Fellowship.

## CONFLICT OF INTEREST STATEMENT

The authors have no conflict of interest to declare.

## Supporting information


Appendix S1.

## Data Availability

The data for this article were from another publication which one of the co‐authors published. These data are already open access and available online at https://www.sciencedirect.com/science/article/abs/pii/S1470160X19305096.
